# Response to “Implementation of a Physician Assistant Emergency Medicine Residency Within a Physician Residency”

**DOI:** 10.5811/westjem.2020.12.51312

**Published:** 2020-12-23

**Authors:** Alina Tsyrulnik, Katja Goldflam, Ryan Coughlin, Jessica Bod, Sharon Chekijian, David Della-Giustina

**Affiliations:** Yale University School of Medicine, Department of Emergency Medicine, New Haven, Connecticut

Dear Editor,

We, the authors of the paper: “Implementation of a Physician Assistant Emergency Medicine Residency Within a Physician Residency” (West J Emerg Med. 2020 Dec 14;22(1):45–8) would like to address concerns raised by members of the emergency medicine (EM) community. Our article describes the successful implementation of a physician assistant (PA) training program within the existing framework of an EM residency. This article was submitted as a “brief educational advance.” It is a description of the logistics of our program and was not powered to draw any statistical conclusions on the limited data of an evaluation tool lacking validation, as was pointed out in the limitations. It does not support or suggest the equivalence of physician graduates of a 3- or 4-year residency in emergency medicine with PA training program graduates. As such, it does not seek to equate the two programs or the skills of their respective graduates, but instead to describe a successful interprofessional educational collaboration.

Further, we want to make it clear that due to our high ED patient volume, including multiple training sites, our physician trainees have not had a decrease in patient or procedure exposure. Our advanced practice provider (APP) trainees present all patients to our senior residents and/or attendings expanding opportunities within our department for interprofessional teamwork. The implementation of a similar program must pay attention to these details in order to ensure optimal training for its physician residents.

This paper is not a commentary on the economics and workforce issues currently facing emergency medicine. Nor is it a substantiation for expanded scope of practice of APPs beyond their intended training. The authors do not wish this work to be used to further political agendas that we do not support. To that end, we would like to explicitly state the following:

1. APPs in emergency medicine should work with the supervision of an EM specialty-trained physician.
2. Patients should be cared for by EM physician-led teams in the emergency department.

We believe in enhancing our residents’ leadership training through their exposure to interprofessional team dynamics while optimizing our APP trainees’ clinical skills in the interest of excellent patient care. We are proud of the collaborative educational programs we have developed and of all our graduates and current trainees.

